# The Effect of Shiatsu Massage on Pain Reduction in Burn Patients

**Published:** 2014-07

**Authors:** Fatemeh Mohaddes Ardabili, Soybeh Purhajari, Tahereh Najafi Ghezeljeh, Hamid Haghani

**Affiliations:** 1 Faculty Member in Medical Surgical Group, School of Nursing and Midwifery, Iran University of Medical Sciences and Health Services, Tehran, Iran;; 2 MSc Student of Nursing, School of Nursing and Midwifery, Iran University of Medical Sciences, Tehran, Iran;; 3 PhD Assistant Professor, Medical- Surgical Group, School of Nursing and Midwifery, Iran University of Medical Sciences, Tehran, Iran;; 4 Department of Statistic and Mathematics, School of Health Management and Information Sciences, Iran University of Medical Sciences, Tehran, Iran

**Keywords:** Burn, Pain, Control, Massage, Shiatsu

## Abstract

**BACKGROUND:**

Burn is a tragedy that follows multiple problems in a patient including pain, anxiety and lack of confidence into medical team. This study evaluated the effect of shiatsu massage on pain intensity of burn patients.

**METHODS:**

A total of 120 burn patients from Motahhari Burn Hospital and of both genders were randomly divided into 4 groups of undergoing hand massage, leg massage, both hand and leg massages, and the control group. The effect of shiatsu massage in pain relief of burned patients was evaluated. The visual analog scale (VAS) was used to assess pain in burn patients.

**RESULTS:**

Pain intensity in the control group before and after the intervention was not statistically significant (*p*=1). In all massage groups, the difference for pain intensity before and after the intervention was statistically significant.

**CONCLUSION:**

According to our data, shiatsu method over both hands and legs were effective in pain reduction and can be recommended together with analgesics to decrease the dose.

## INTRODUCTION

Burn is a tragedy with many problems such as pain^1^ and due to severe tissue damages and psychological problems, the patient requires special assistance to alleviate the injuries.^2^ It is a major cause of death, dis­ability and high cost in health care and during pregnancy can increase the mortality and morbidity more in both mother and infant.^3^^,^^4^ In burn patients, *Pseudomonas aeruginosa* is an important cause of nosocomial infection that may cause septicemia and death denoting to its public health importance more and more.^5^ Application of topical anti-bacterial agents and disinfectants such as silver sulfadiazine (SSD) was shown as the most widely used topical therapy in burn injuries with anti-microbial effects.^6^.Herbal medicines with less toxicity and as inexpensive therapies have been used in healing of burn injuries,^7^^-^^11^ but reports on pain control in burn patients is very few. 

A common method of pain control in burned patients is use of narcotic analgesics, even the use of narcotic analgesics alone cannot completely relieve the pain in burn patients.^12^ Tranquilizers that are most often prescribed by physicians may be associated with side effects such as respiratory system suppression and drowsiness.^13^ One of the methods in reducing pain was shown to be the use of massage.^14^ as a complementary therapy reducing the need for analgesic drugs and also limit the side effects in burn patients. 

Shiatsu as a complementary massage therapy is the pressure and scrubbing of the energy pathways in the body based on knowledge and application of energy to treat and relieve pain in any part of the underlying diseases.^15^ This study evaluates the effect of shiatsu massage on pain intensity in burn patients.

## MATERIALS AND METHODS

The study population was consisted of 120 burn patients from Motahhari Burn Hospital and of both genders. They were randomly divided into 4 groups of undergoing hand massage, leg massage, both hand and leg massages, and the control who did not receive any massage. The effect of shiatsu massage in pain relief of burned patients was evaluated as described before.^15^ In all massage groups, the patients were placed on a bed or chair in a comfortable position and received the massage for 20 minutes. During the massage, the patients closed their eyes and focused on the procedure as reported before.^16^ The visual analog scale (VAS) was used to assess pain in burn patients as described before.^17^ In all groups, data collection was done before and after the intervention. Data analysis was performed using SPSS software (Version 19, Chicago, IL, USA) and paired t-test. A *p* value less than 0.05 was statistically considered significant.

## RESULTS

**Fig. 1:**
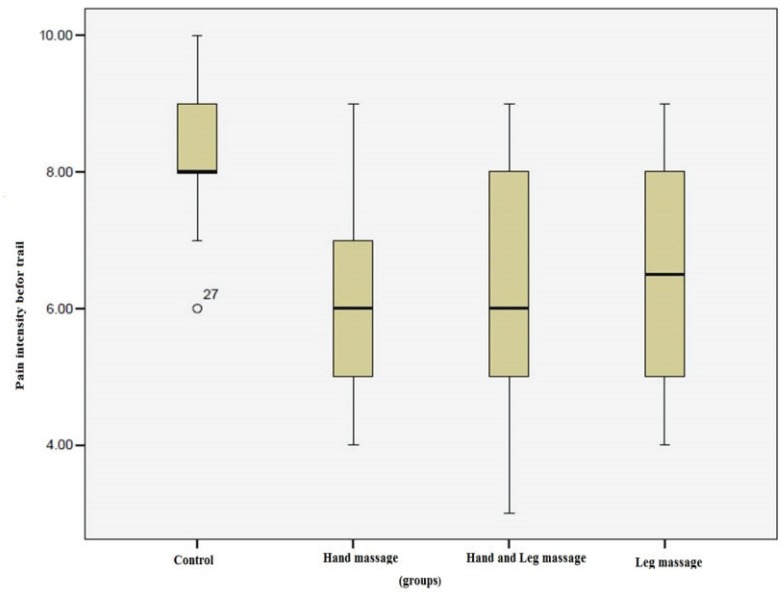
Pain intensity distribution in the four groups before intervention

**Fig. 2:**
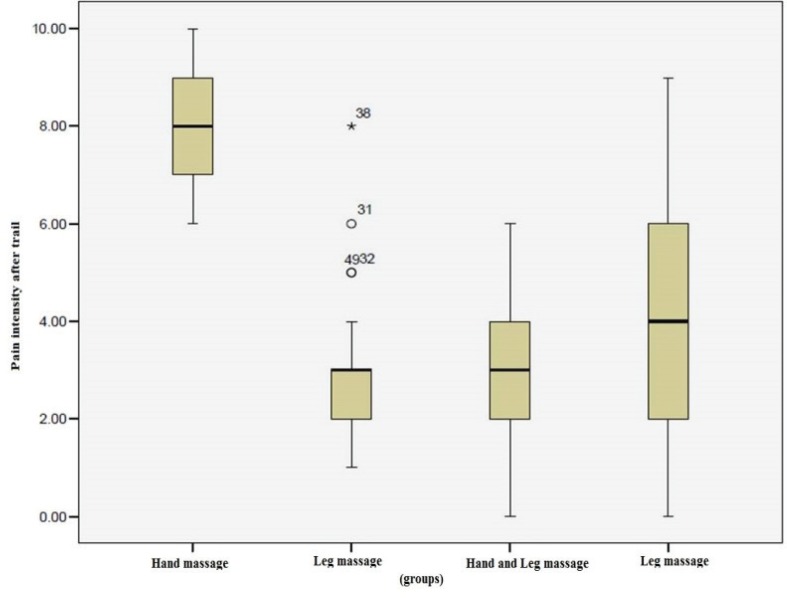
Pain intensity distribution in the four groups after intervention

Pain intensity in the control group before and after the intervention was not statistically significant (*p*=1). In all massage groups, the difference for pain intensity before and after the intervention was statistically significant (*p*=0.001) while the pain intensity before the intervention was higher. Distribution of pain intensity score before massage was higher in control group in comparison to other groups (Figure 1). After massage, the distribution of pain intensity score was higher in all massage groups in comparison to the control group, but after massage; in massage groups, the score of pain intensity decreased (Figure 2). 

## DISCUSSION

Our findings showed no significant difference between massage groups and control group regarding pain severity before massage. There was a significant difference between massage groups and control group regarding pain severity after massage. So massage may be an important therapy in management of pain in burned patients and shiatsu can be a model for future researches in complementary medicine in primary care system. Leg and hand massage was more effective in burn pain control in comparison to leg and hand massage only. In reducing pain, other researchers reported the efficacy of massage in pain reduction.^14^^,^^15^

So we can suggest a 20 min hand, leg, hand and leg massage intervention in conjunction with analgesics to control the pain in burn patients decreasing the needed dose of analgesics.

## CONFLICT OF INTEREST

The authors declare no conflict of interest. 

## Footenote

Please cite this paper as:

Mohades Ardabili F, Purhajari S, Najafi T, Haghani H. The Effect of Shiatsu Massage on Pain Reduction in Burn Patients. World J Plast Surg 2014;3(2):115-118.
